# Huaier extract synergizes with tamoxifen to induce autophagy and apoptosis in ER-positive breast cancer cells

**DOI:** 10.18632/oncotarget.8303

**Published:** 2016-03-23

**Authors:** Wenwen Qi, Mingjuan Sun, Xiangnan Kong, Yaming Li, Xiaolong Wang, Shangge Lv, Xia Ding, Sumei Gao, Jinjing Cun, Chang Cai, Xiaoting Wang, Junfei Chen, Aijun Yin, Qifeng Yang

**Affiliations:** ^1^ Department of Breast Surgery, Qilu Hospital, Shandong University, Jinan, P.R. China; ^2^ Shandong Cancer Hospital and Institute, Jinan, P.R. China; ^3^ Laboratory Medicine Center, Qilu Hospital of Shandong University, Qingdao, P.R. China; ^4^ Department of Oncology, Qilu Hospital, Shandong University, Jinan, P.R. China; ^5^ Department of Obstetrics and Gynecology, Qilu Hospital, Shandong University, Jinan, P.R. China; ^6^ Pathology Tissue Bank, Qilu Hospital, Shandong University, Jinan, P.R. China

**Keywords:** Huaier extract, tamoxifen, ER-positive breast cancer, autophagy, apoptosis

## Abstract

Tamoxifen (TAM) is the most widely used endocrine therapy for estrogen receptor (ER)-positive breast cancer patients, but side effects and the gradual development of insensitivity limit its application. We investigated whether Huaier extract, a traditional Chinese medicine, in combination with TAM would improve treatment efficacy in ER-positive breast cancers. MTT, colony formation, and invasion and migration assays revealed that the combined treatment had stronger anticancer effects than either treatment alone. Huaier extract enhanced TAM-induced autophagy, apoptosis, and G0/G1 cell cycle arrest, as measured by acidic vesicular organelle (AVO) staining, TUNEL, flow cytometry, and western blot. Additionally, combined treatment inhibited tumorigenesis and metastasis by suppressing the AKT/mTOR signaling pathway. Huaier extract also enhanced the inhibitory effects of TAM on tumor growth *in vivo* in a xenograft mouse model. These results show that Huaier extract synergizes with TAM to induce autophagy and apoptosis in ER-positive breast cancer cells by suppressing the AKT/mTOR pathway.

## INTRODUCTION

Selective estrogen receptor modulators (SERMs) prevent estrogen from binding to estrogen receptors. The SERM tamoxifen (TAM) has been used as the standard adjuvant endocrine treatment for more than 30 years for all stages of estrogen-dependent breast cancer [1]. A large randomized clinical trial found that 5 years of treatment with TAM reduced the incidence of invasive breast cancer by about 50% in postmenopausal women who were at high risk of BC [2]. However, treatment with TAM alone for estrogen-dependent breast cancer during the premenopausal period is associated with an increased risk of blood clots, especially in the lungs and legs [3], stroke [4], cataracts [5], and endometrial and uterine cancers [4, 6]. Patients also become insensitive to this treatment after extended TAM administration and experience other side effects. Combination therapy might enhance the effects of TAM in ER-positive BCs and reduce adverse side effects by decreasing the therapeutic dose of TAM [7].

Among combination therapies, traditional Chinese medicine (TCM) has gained popularity for its ability to kill tumor cells while reducing harm to normal cells. In addition, TCM herbal treatment has a low economic cost and increases chemotherapy efficiency, reduces toxicity, prolongs survival time, and improves quality of life and immune functions [8]. *Trametes robiniophila murr* (Huaier) is a type of fungus from China which has been used in TCM for approximately 1600 years. It is isolated from the extract of the officinal fungi, and proteoglycan has been identified as the effective ingredient (containing 8.72% water, 12.93% amino acids and 41.53% polysaccharides) [9]. Huaier extract has been studied extensively for its antitumor effects, including inhibition of cell proliferation [10], anti-metastasis [11], interference with tumor angiogenesis [12], induction of autophagic cell death [13], and tumor-specific immunomodulatory effects [14, 15]. Our study demonstrates for the first time that Huaier extract synergizes with tamoxifen to induce autophagy and apoptosis in ER-positive breast cancer cells by inhibiting the AKT signaling pathway. These effects support the use of Huaier extract in combination with TAM for treating ER-α-positive breast cancer.

## RESULTS

### HTA 2.0 microarray assay revealed key pathways regulated by Huaier extract

**Figure 1:**
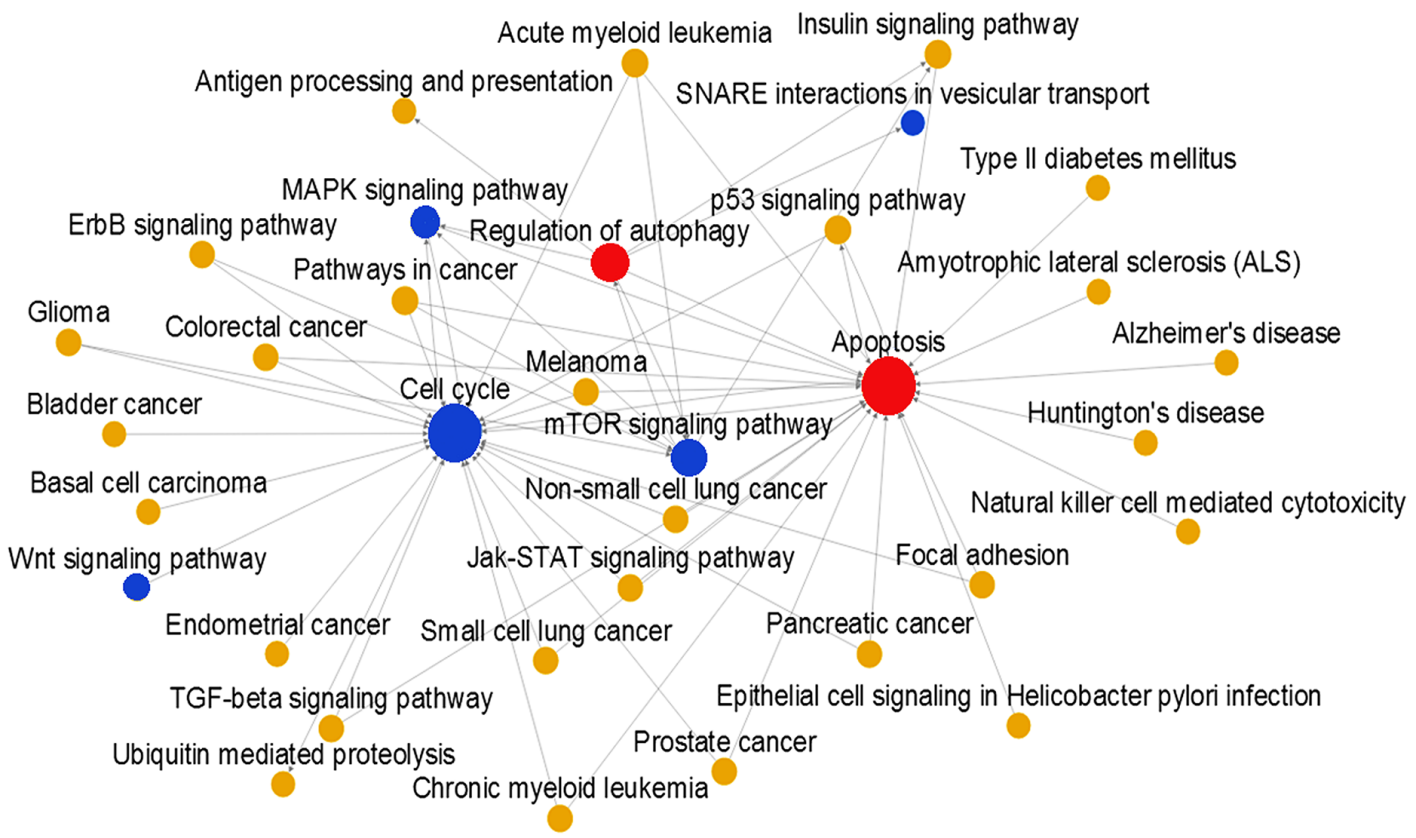
Signal pathway relation network in MCF-7 cells Red indicates upregulated, blue indicates downregulated, and yellow indicates unchanged pathways. The area of the circles indicates the value of betweenness centrality. Line segments indicate pathway-pathway interactions.

Based on methods described previously, the HTA 2.0 microarray assay was used to construct a pathway-pathway interaction network (Figure 1). Pathways of interest were closely connected, and most were located in the center of the network. Red indicates upregulated, blue indicates downregulated, and yellow indicates unchanged pathways. The area of the circles indicates the value of betweenness centrality. Huaier extract activated autophagy and apoptosis pathways and inhibited the cell cycle and mTOR pathway.

### The combination of Huaier extract and TAM reduced the viability and motility of ER-positive breast cancer cells

**Figure 2:**
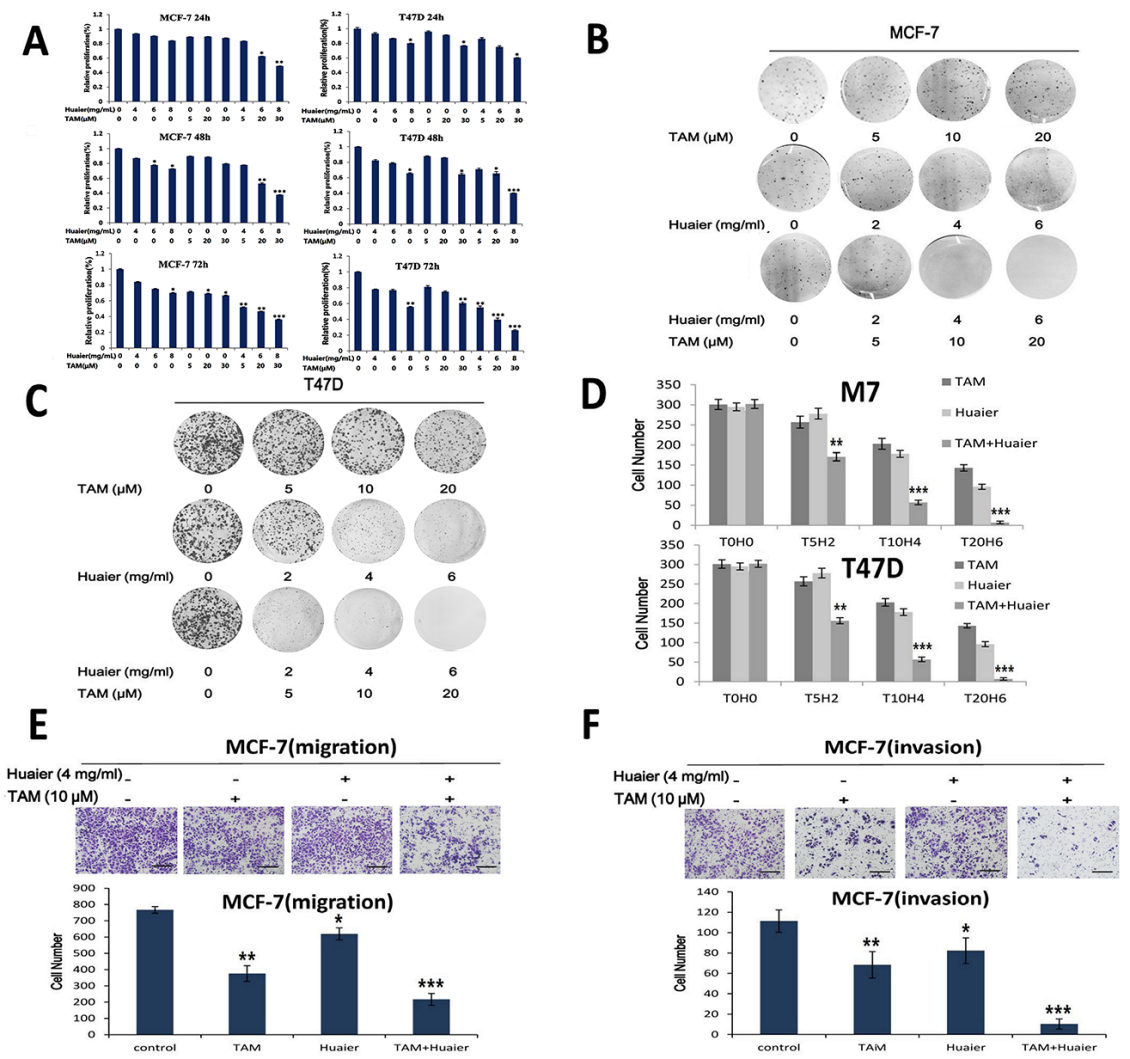
Combined treatment reduced cell viability and motility more than monotherapies **A.** The effect of Huaier extract and TAM on cell viability was measured by MTT assay as described in Materials and Methods. Combined treatment inhibited viability in both cell lines in a dose- and time-dependent manner. MCF-7 **B.** and T47D **C.** cells formed colonies. Representative Giemsa staining pictures of the colonies are shown. MCF-7 and T47D cell colonies **D.** were counted. Cell mobility was strongly inhibited by combined treatment as shown by migration **E.** and invasion **F.** assays using the Transwell system. Giemsa was used as a staining agent and cell numbers were counted in six representative fields. Bars, 50 μm. All experiments were performed in triplicate and data are presented as the mean ± SD of three separate experiments (*p < 0.05; **p < 0.01; ***p < 0.001 vs. the control group).

An MTT assay was used to measure cell viability. As shown in Figure 2A, combined therapy with Huaier and TAM significantly reduced the viability of both MCF-7 and T47D cells in a time- and dose-dependent manner. Cell viability decreased sharply after administration of 4 mg /mL Huaier with 5 μM TAM, independent of the treatment time. A colony formation assay revealed that combined treatment decreased the proliferation rate of both MCF-7 and T47D cells (Figure 2B, 2C and 2D).

Migration and invasion assays were carried out to measure cell motility. As indicated in Figure 2E and 2F, the combination of 4 mg/mL Huaier extract and 10 μM TAM inhibited migration and invasion in MCF-7 cells more than single drug treatments.

### Huaier extract synergizes with tamoxifen to induce autophagic cell death in ER-positive breast cancer cells

**Figure 3:**
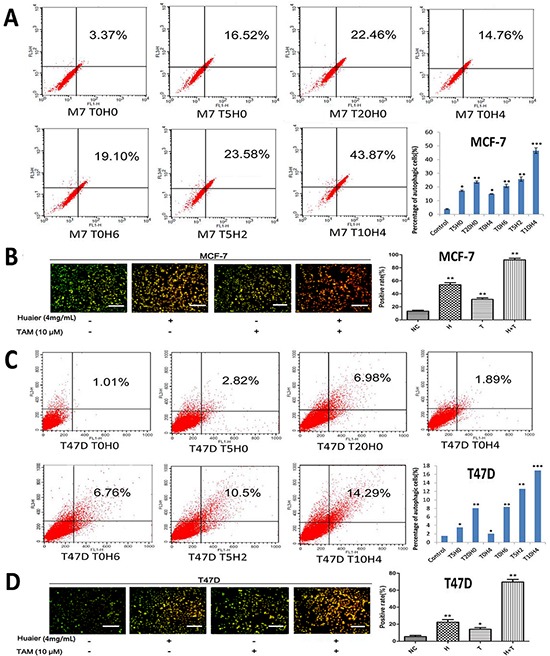
Huaier extract synergizes with tamoxifen to induce autophagy in ER-positive breast cancer cells MCF-7 **A.** and T47D **C.** cells were labeled with Acridine orange (AO) after the indicated treatments and quantified using flow cytometry. FL1-H indicates green color intensity (cytoplasm and nucleus), whereas FL3-H shows red color intensity (AVO). Cells in up quadrants were considered AVO-positive. Treated MCF-7 **B.** and T47D **D.** cells were stained with AO and examined under a fluorescence microscope. Bars, 50 μm. All of the experiments were performed in triplicate and data are presented as the mean ± SD of three separate experiments (*p < 0.05; **p < 0.01; ***p < 0.001 vs. the control group).

To quantify autophagic cell death in cells treated with Huaier extract, TAM, or both, we used flow cytometry analysis (Figure 3A and 3C) and an AVO staining assay (Figure 3B and 3D) [13]. As shown in Figure 3, both Huaier extract and TAM induced autophagic cell death. Combining the two treatments induced the formation of more autophagosomes than either of the drugs alone.

### Huaier extract synergizes with tamoxifen to induce apoptosis in ER-positive breast cancer cells

**Figure 4:**
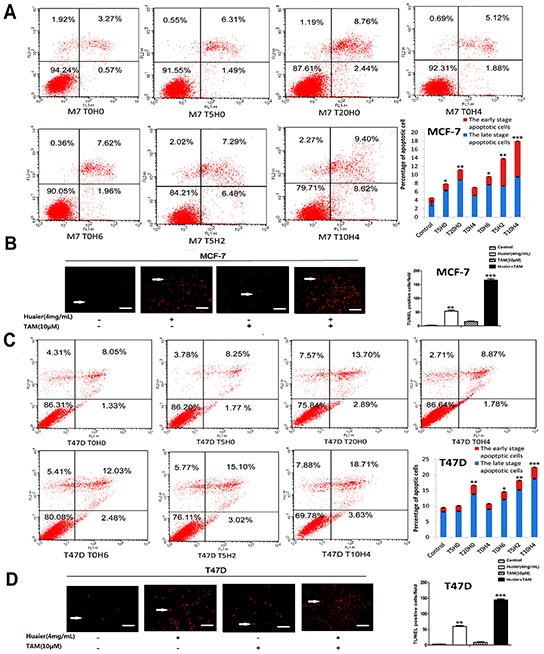
Huaier extract synergizes with tamoxifen to induce apoptosis in ER-positive breast cancer cells Flow cytometric analysis of PI-Annexin-V to quantify apoptosis in MCF-7 **A.** and T47D **C.** cells after treatment as described in Materials and Methods. Representative TUNEL staining (red fluorescence) of MCF-7 **B.** and T47D **D.** cells treated with Huaier (0 or 4mg/ml) and TAM (0 or 10μmol/L). Bars, 50 μm. Columns are the average of three independent experiments. (*p < 0.05; **p < 0.01; ***p < 0.001 vs. the control group).

We used the TUNEL assay to detect the modes of cell death induced by Huaier extract and TAM (Figure 4B and 4D). As shown in Figure 4, Huaier extract induced necrosis and apoptosis, which was consistent with our previous data [9]. Huaier extract also synergized with tamoxifen to induce autophagy and apoptosis in ER-positive breast cancer cells. Additionally, intact cells, early apoptotic cells, and late apoptotic or dead cells can be identified using PI-annexin-V double staining [16]. This method showed that after combined drug treatment, late apoptosis or cell death rates and early apoptosis rates increased in a dose-dependent manner in both MCF-7 and T47D cells (Figure 4A and 4C).

### Huaier extract synergizes with tamoxifen to induce cell-cycle arrest in ER-positive breast cancer cells

**Figure 5:**
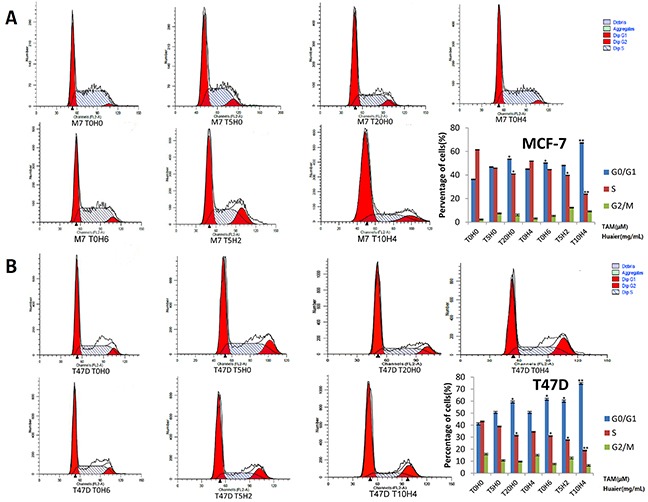
Cell-cycle arrest at G0 / G1 in response to Huaier and/or TAM treatment **A.** Huaier (0, 2, 4, or 6mg/ml) or TAM (0, 5, 10, or 20μmol/L)-induced G0 / G1 arrest in MCF-7 cells and cell cycle distribution in MCF-7 cells was assessed by flow cytometry after staining with propidium iodide (PI). **B.** Arrest at G0 / G1 in T47D cells was induced by the same treatment used in MCF-7 cells. All of the experiments were performed in triplicate and data are presented as the mean ± SD of three separate experiments (*p < 0.05; **p < 0.01; ***p < 0.001 vs. the control group).

Cell-cycle distribution was analyzed by flow cytometry to determine the inhibitory effect of Huaier and TAM. MCF7 and T47D cells were exposed to Huaier extract, TAM, or both for a total of 48 h. As shown in Figure 5, G0/G1 arrest increased in cells exposed to these drugs compared to untreated cells. All treatments also concomitantly decreased the proportion of cells in the S phase. These results revealed that both Huaier extract and TAM slowed MCF-7 (Figure 5A) and T47D (Figure 5B) cell proliferation via cell-cycle arrest at the G0/G1 phase, which is consistent with previous studies [9, 17]. Furthermore, after combined drug treatment, the percentage of cells in the G0/G1 phase increased dramatically in a dose-dependent manner in both MCF-7 and T47D cells.

### TAM and Huaier extract target AKT in ER-positive breast cancer cells

**Figure 6:**
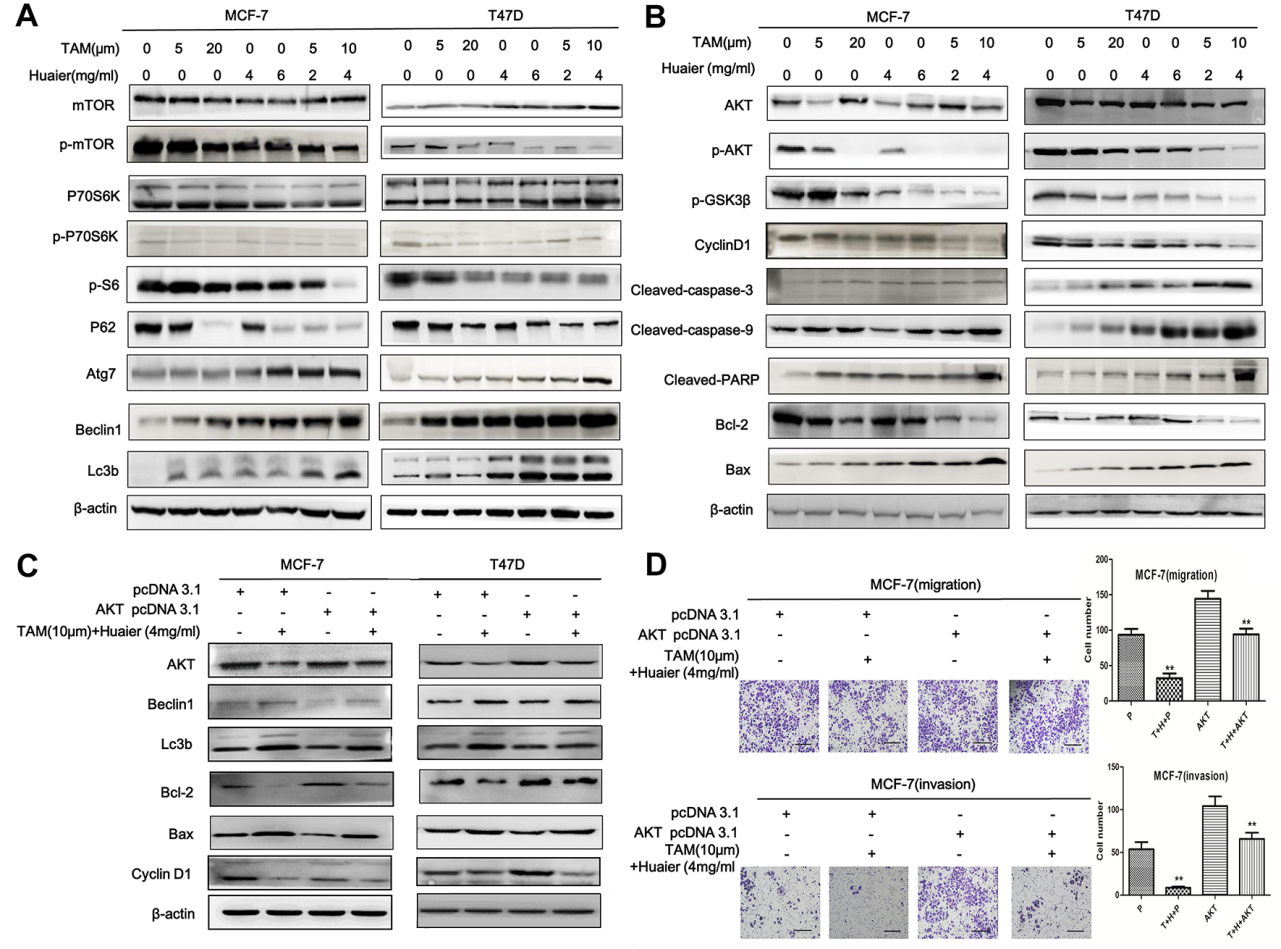
Huaier extract synergized with tamoxifen in signal pathway induction in ER-positive breast cancer cells **A.** Combined treatment induced more autophagy than either monotherapy (mTOR, p-mTOR, P70S6, p-P70S6, p-S6, P62, Atg7, Beclin1, and Lc3b). The autophagy effect was stronger in T47D than in MCF-7 cells. **B.** Western blot analysis revealed that Huaier extract synergizes with tamoxifen to induce apoptosis in ER-positive breast cancer cells (Cleaved-capase-3, Cleaved-capase-9, Cleaved-PARP, Bcl-2, and BCL2-associated X protein (BAX) proteins) and G0/G1 arrest (p-GSK3β, Cyclin D1). **B, C, D.** AKT is a target of the combination treatment. **B.** Effects of Huaier extract or/and TAM on expression of AKT and p-AKT proteins. **C.** Western blot analysis demonstrated that the transfection of AKT pcDNA3.1 reversed the protein changes induced by combined treatment (Beclin1, Lc3b, Bcl-2, Bax, and Cyclin D1). Expression of β-actin was used as an internal control in all above analyses. **D.** There was no significant difference in migration or invasion between AKT pcDNA3.1 with combined treatment and pcDNA3.1 without treatment groups. Bars, 50 μm. The data represent average cell numbers from at least 10 viewing fields. All results shown are from one of three independent experiments performed (*p < 0.05; **p < 0.01; ***p < 0.001 vs. the control group).

MCF-7 and T47D cells were treated with Huaier extract (0, 4, or 6 mg/mL), TAM (0, 5, or 20 μM), or both (Figure 6A–6D) for 48 hours. As shown in Figure 6A and 6B, levels of proteins involved in the AKT/mTOR pathway (AKT, p-AKT, mTOR, p-mTOR, p70S6K, p-p70S6K), cell autophagy (p-S6, P62, Atg7, Beclin1, Lc3b), and apoptosis (Cleaved-caspase-3, Cleaved-caspase-9, Cleaved-PARP, Bcl-2, Bax) were all altered by treatment. Expression values were normalized to controls.

Because AKT induces changes in cell cycle distribution [18], we measured the effects of treatment on the levels of select cell cycle regulatory proteins (Figure 6B). Cyclin D1 stimulates the G1-S checkpoints, and the role of p-GSK3β as an upstream regulator of Cyclin D1 [19] was consistent with the flow cytometry analysis. Taken together, these findings indicate that AKT is a likely target of synergistic treatment with TAM and Huaier extract in ER-positive breast cancer.

To further confirm this finding, we investigated the effects of AKT overexpression in ER-positive breast cancer cells. Western blot analysis confirmed that AKT overexpression reduced levels of Bax, Beclin1, and Lc3b protein and partially reversed the suppression of Bcl-2 in MCF-7 and T47D cells after combined treatment (Figure 6C). The levels of these proteins did not differ between the group transfected with pcDNA 3.1 without drug treatment and the group transfected with AKT pcDNA 3.1 with drug treatment. AKT pcDNA 3.1 transfection together with combined drug treatment did not alter cellular migration or invasion as compared to pcDNA 3.1 transfection without drug treatment in MCF-7 cells (Figure 6). These results confirm that AKT is a down-stream target of combined TAM and Huaier extract treatment in ER-positive breast cancer.

### Combined TAM and Huaier extract treatment inhibited the growth of subcutaneous tumors

**Figure 7:**
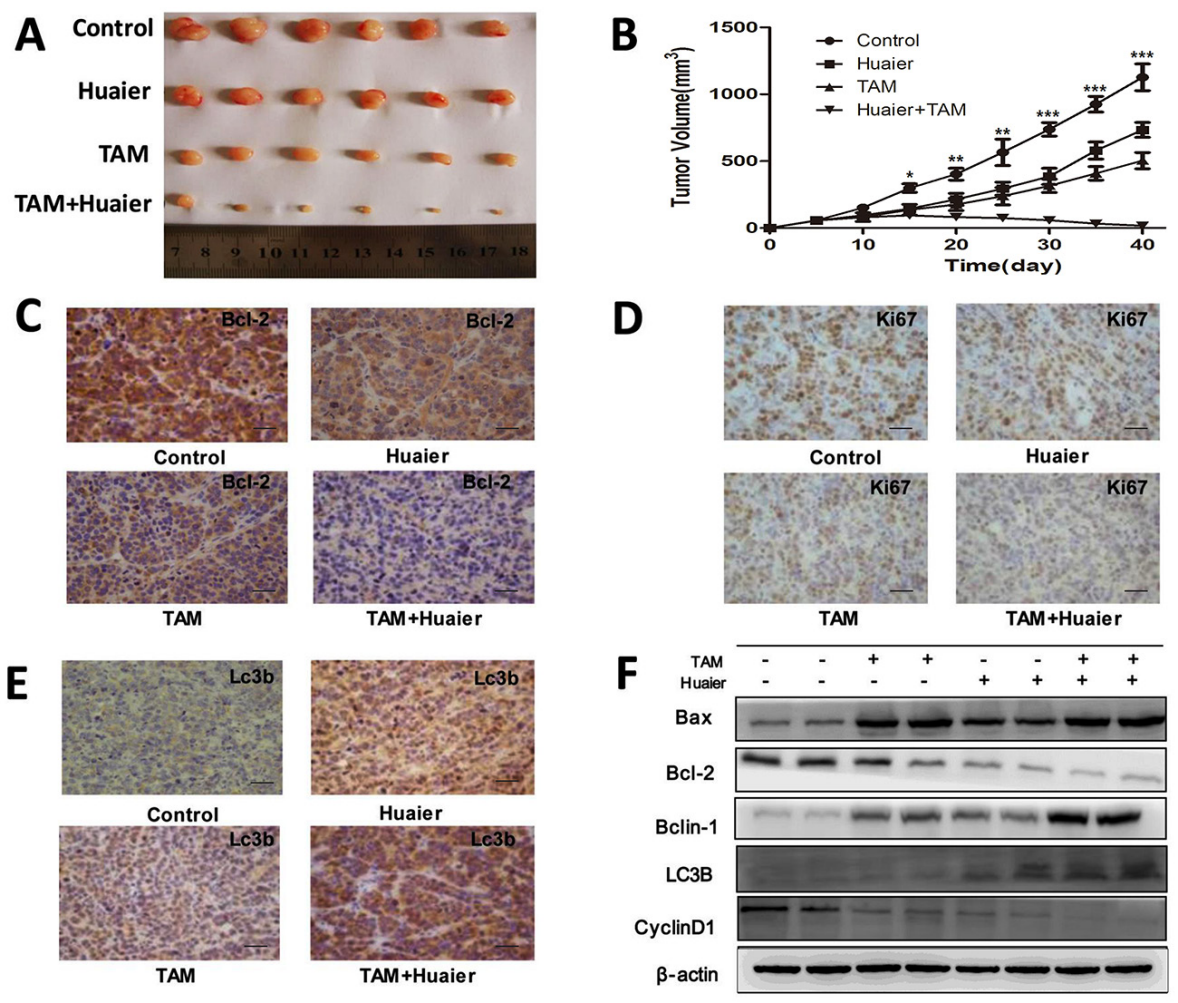
Combined treatment enhanced the suppression of tumorigenesis in a xenograft model **A.** Flank tumors were established in BALB/c nu/nu female mice as described in Materials and Methods. Mice were killed 40 days after flank injection; representative tumors are shown. **B.** Growth curves of xenograft tumors after the injection of MCF-7 cells. Mean tumor volumes ± SD are shown. **C.** Immunohistochemical analysis of Bcl-2(C), Ki67 **D.** and Lc3b **E.** from the collected xenografts. Bars, 50 μm. **F.** Combined drug treatment increased levels of Bax, Beclin-1, and Lc3b and decreased Bcl-2 and Cyclin D1 levels compared to the control and monotherapy groups. The experiments were performed in triplicate and data are presented as the mean ± SD of three separate experiments (*p < 0.05; **p < 0.01; ***p < 0.001 vs. the control group).

In order to demine whether the curative effect of combination treatment was greater than either monotherapy *in vivo*, MCF-7 breast cancer cells were subcutaneously injected into the right flank of BALB/c nu/nu mice. As shown in Figure 7A, xenograft tumor growth was reduced in the combined treatment group as compared to the control group and both monotherapy groups. On day 40, tumors in the combined treatment group (17.41 ± 8.99 mm^3^) were smaller than those in the Huaier treated (733.45 ± 55.93 mm^3^), TAM treated (503.844 ± 61.12 mm^3^), and untreated control (1127.40 ± 100.07 mm^3^) groups (Figure 7B). In order to examine the mechanism underlying the inhibition of tumor growth by Huaier extract and TAM *in vivo*, we measured Lc3b, Bcl-2, and Ki67 protein levels using immunohistochemical staining. As shown in Figure 7, Bcl-2 and Ki67 levels decreased and Lc3b levels increased in the combined treatment group compared to the control group and monotherapy groups. Furthermore, combined treatment increased Bax, Beclin-1, and Lc3b levels and decreased Bcl-2 and Cyclin D1 levels compared to the control and monotherapy groups *in vivo*. These results suggest that Huaier extract synergizes with tamoxifen to induce autophagy and apoptosis in ER-positive breast cancer cells *in vivo*.

## DISCUSSION

TAM is widely used for treating ER-positive BCs, but its side effects and the gradual development of insensitivity limit its effectiveness. Many studies have shown that resistance to TAM is partly mediated through the AKT pathway, which promotes estrogen-independent cell proliferation [20]. The AKT pathway also plays a crucial role in breast cancer pathogenesis, and AKT upregulation is associated with more aggressive clinical phenotypes and worse outcomes in endocrine-treated patients [21, 22]. In this study, we demonstrate for the first time that Huaier extract, which has long been used in traditional Chinese medicine, synergizes with TAM to increase autophagy and apoptosis in ER-positive breast cancer cells by inhibiting the AKT/mTOR signaling pathway.

In our previous studies, we have shown that Huaier extract exerts a strong anti-proliferative effect by inducing caspase-dependent apoptosis, increasing autophagic cell death, suppressing the estrogen receptor α pathway, and inhibiting angiogenesis in both ER-positive and ER-negative breast cancers [9, 12, 23]. In this study, we found that Huaier extract synergizes with TAM to increase ER-positive breast cancer cell autophagy and apoptosis. As shown in the MTT and the colony formation assays, combined treatment had a stronger cytotoxic effect on MCF-7 and T47D cells than treatment with either drug alone. In addition, combined treatment suppressed motility more effectively in ER-positive breast cancer cells as demonstrated by migration and invasion assays.

In the last decade, it has been well established that programmed cell death (PCD) is not confined to apoptosis (type-I PCD), but instead is involved in various mechanisms of active self-destruction. One such mechanism is autophagy (also called type-II PCD), which is characterized by distinct morphological and biochemical features. [24–26]. Recent studies suggest that inducing autophagy is a promising new strategy for fighting human diseases, including cancers [27, 28]. During autophagic cell death, portions of the cytoplasm are sequestered into double membrane vesicles known as autophagosomes, which then fuse with lysosomes to form single-membrane autophagolysosomes. Autophagic cells are thus identified by the accumulation of vacuoles and LC3, which forms the membrane of the autophagosome [29]. In this study, we examined Hauier extract and TAM-induced autophagy in breast cancer cells using an acidic vesicular organelles (AVO) staining assay and flow cytometry analysis. LC3B, Beclin1, and Atg7 levels indicate that the induction of autophagy is dose-dependent, and combined treatment therapy enhanced TAM-induced increases in autophagy. Moreover, the PI-Annexin-V staining assay, TUNEL assay, and western blot analysis confirmed the presence of cell apoptosis (type-I PCD) in both cell lines that received treatment. The mitochondrial pathway is an important mediator of cell apoptosis in mammals. In the mitochondrial pathway, Bcl-2 family members, including the anti-apoptotic Bcl-2 protein and pro-apoptotic Bax protein, regulate apoptosis in different situations [30]. Bax assists in the release of cytochrome c from the mitochondrial intermembrane space into the cytosol by increasing the permeability of the outer membrane. The combined treatment therapy increased Bax protein levels compared to the monotherapy groups, indicating that Huaier extract synergizes with tamoxifen to induce autophagy in ER-positive breast cancer cells. Thus, both autophagy and apoptosis played important roles in combined drug treatment-induced PCD.

Earlier studies also demonstrated that TAM induces arrest in the G0/G1 phase in breast cancer cells [17]; here, we demonstrated for the first time that Huaier extract has similar effects using flow cytometry analysis. Cyclin D1 is important for inducing S phase entry, and inhibition of Cyclin D1 induces cell cycle arrest in the G1 phase [31]. Flow cytometry and analysis of Cyclin D1 expression revealed that the combination of Huaier extract and TAM enhanced cell cycle arrest.

Gene mutations and copy number alterations (CNAs) activate the AKT/mTOR pathway in many tumor types [32], and numerous studies demonstrate that mTOR kinase suppresses autophagy and apoptosis [33]. On one hand, mTOR suppresses autophagy through the Atg proteins by interfering with the formation of autophagosomes [34]. Our results show that combined treatment dramatically decreases mTOR activity. This inhibition resulted in reduced phosphorylation of the mTOR downstream targets P70S6K, S6, and P62. These findings indicate that combined treatment induced autophagy in breast cancer cells at least partly by inhibiting the mTOR/S6K signaling pathway. On the other hand, mTOR also suppresses apoptosis through the caspases, a group of cysteine-aspartic proteases [35]. The initiator caspases (e.g. caspase-9), which were activated by apoptotic stimulation, cleaved and activated executioner caspases (e.g. caspase-3). This caspase cascade leads to the cleavage of PARP, a DNA repair enzyme and known caspase substrate, and eventually to DNA degradation. Caspase activation, which was measured by examining protein levels and using a TUNEL assay, also played a role here following the combined drug treatment. Furthermore, cell cycle arrest may be crucial to the fragmentation of DNA, which is either repaired or leads to death [36]. Inactivation of the DNA repair enzyme PARP renders cells unable to repair fragmented DNA, eventually leading to the arrest of cell cycle progression at the G0/G1 phase. Glycogen synthase kinase-3 (GSK3) is also a critical downstream element of the AKT/mTOR pathway. Here, inhibiting AKT/mTOR decreased GSK3β and Cyclin D1 expression, resulting in G0/G1 cell cycle arrest. However, overexpression of AKT only partially reversed the decrease in expression of BCL2/Lc3b/Cyclin D1 following Huaier extract treatment, so there are likely other functional mediators of Huaier's effect on endocrine therapy resistance. The signal pathway analysis from the HTA 2.0 transcriptome microarray assay was also consistent with these data.

In summary, our results reveal for the first time that Huaier extract synergizes with tamoxifen to increase autophagy, apoptosis, and G0/G1 arrest in ER-positive breast cancer cells. These results suggest that this combined treatment may be more effective than monotherapy and might help reduce side effects in patients with ER-positive breast cancer. Future studies should examine treatment using Huaier extract both alone and in combination with radiation or chemotherapy in a clinical setting. Additionally, the effects of Hauier extract both alone and in combination with other drugs on deeper molecular mechanisms involved in cancer, such as lncRNA and miRNA, should be examined.

## MATERIALS AND METHODS

### Cell lines and reagents

ER-positive breast cancer cell lines MCF-7 and T47D were obtained from American Type Culture Collection (ATCC; Rockville, MD, USA). Huaier extract was kindly provided by Gaitianli Medicine Co., Ltd. (Jiangsu, China) and prepared as described before [9]. Tamoxifen was purchased from Sigma-Aldrich (St. Louis, MO, USA). Anti-Bcl-2, Ki67, ERα, and BAX were purchased from Dako Corp. (Carpinteria, CA, USA). Mouse monoclonal antibody against β-actin was purchased from Sigma-Aldrich (St. Louis, MO, USA). All of the remaining antibodies were obtained from Cell Signaling Technology (Beverly, MA, USA), unless otherwise specified.

### Cell culture

MCF-7 cells were routinely maintained in DMEM medium (Gibco-BRL, Rockville, IN, USA) supplemented with 10% fetal bovine serum (FBS, Haoyang Biological Manufacture Co. Ltd., Tianjin, China), 100 U/mL penicillin, and 100 μg/mL streptomycin. T47D cells were cultured in RPMI-1640 medium (Gibco-BRL) with 10% FBS and 10 μg/mL bovine insulin (Sigma-Aldrich, St. Louis, MO, USA). MCF-7 and T47D cells were routinely cultured at 37°C with 5% CO_2_ in a humidified incubator.

### HTA 2.0 microarray assay and pathway analysis

Total RNA was isolated from MCF-7 cells with TRIzol (Invitrogen, Carlsbad, CA, USA) after 72 h of treatment with Huaier aqueous extract (8 mg/Ml with DMEM). Equal amounts of RNA were pooled from three donors for each sample. Biotinylated cDNA was prepared from 250 ng total RNA according to the Affymetrix standard protocol using the Ambion® WT Expression kit. cDNA was hybridized in a Hybridization Oven 645 for 16 h at 45°C on a GeneChip Human Transcriptome Array 2.0 (HTA 2.0). GeneChips were scanned using the Affymetrix® GeneChip Command Console (AGCC). The data were analyzed using the Robust Multichip Analysis (RMA) algorithm with Affymetrix default analysis settings and global scaling as a normalization method. Fold change was used to identify differentially expressed genes (DEGs). Pathway analysis was used to find significant pathways based on differential gene expression according to KEGG, Biocarta, and Reatome. Fisher's exact test and χ^2^ tests were employed to select significant pathways, and the threshold of significance was defined by *P*-value and false discovery rate (FDR).

### Plasmid construction and transfection

For AKT overexpression, AKT cDNA was cloned into the multiple cloning site of the pcDNA3.1 vector (Invitrogen, Carlsbad, CA, USA). The expression plasmid vector and the empty vector were used to transfect MCF-7 and T47D cells using lipofectamine 2000 (Thermo Fisher Scientific Inc., Waltham, MA, USA) to establish AKT overexpression (AKT-OVER) and control (Control) cell lines.

### Cell viability assay

Cell viability was determined by a 3-(4, 5-dimethylthiazol-2-yl)-2, 5-diphenyltetrazolium bromide (MTT) assay. MCF-7 (3×10^3^ cells/well) and T47D (1.5×10^3^ cells/well) cells were cultured in 96-well plates. After incubation overnight, the medium was replaced with solutions containing different concentrations of Huaier (0, 4, 6, or 8 mg/mL) and TAM (0, 5, 20, or 3 μM), followed by incubation for 24, 48, or 72 h. Afterwards, 20 μL of MTT (5 mg/mL in PBS) was added to each well and the cells were incubated for another 4 h at 37°C. The supernatants were then aspirated carefully and 100 μL of dimethyl sulfoxide (DMSO) was added to each well. The plates were shaken for an additional 10 min and the absorbance values were read using a Microplate Reader (Bio-Rad, Hercules, CA, USA) at 570 nm [37].

### Migration and invasion assays

Migration and invasion assays were performed as described previously [38] using the Transwell system (Corning Costar, Lowell, MA, USA). In the migration assay, cells were starved in serum-free medium for 12 h at 37°C. 700 μL of medium with 20% FBS was added to the lower well of each chamber and 1×10^5^ cells suspended in serum-free medium were added to the upper inserts. After incubation for 48h, the total number of cells adhering to the lower surface of the membrane was quantified in six representative fields. The invasion assay was performed in the same way as the migration assay except that the membrane was coated with matrigel (BD Biosciences, Bedford, MA, USA).

### Colony forming assay

MCF-7 and T47D cells were seeded (1×10^3^ cells/well) in six well plates. Cells were allowed to grow for 24 h. After 24 h, cells were treated with Huaier extract and/or tamoxifen for 48 h. Then medium was replaced and cells were allowed to grow for 10 days. Thereafter, cells were washed, fixed and stained with 1% crystal violet solution. Excess staining was washed with PBS and images were acquired using a microscope.

### Immunocytochemical analysis

Acridine Orange staining was performed as previously described in [39]. MCF-7 and T47D cells were seeded in six-well plates on a coverslip and then treated with Huaier extract or/and tamoxifen. Cells were fixed with 100% cold methanol and then stained with 0.01% Acridine Orange. The slides were sealed by neutral resin and observed under microscope at a magnification of ×400. The images were analyzed using ImageJ software.

### Flow cytometry analysis of acidic vesicular organelles (AVO)

In order to quantify the change in the number of AVOs in cells treated with Huaier extract, cells were stained with Acridine orange (1 μg/mL) in PBS at 37°C for 15 min in the dark. The cells were washed with PBS twice and then suspended in PBS for immediate analysis. The data were analyzed using BD Cell Quest software.

### Identification of apoptosis by PI-Annexin-V staining

This assay was performed to detect cell apoptosis with an Annexin V-FITC Apoptosis Detection Kit (JingMei Biotech, Beijing, China), following the instructions from the manufacturer. In brief, harvested cells were suspended in 100 μL binding buffer to achieve a concentration of 1×10^6^/mL. Then, 5 μL Annexin V-FITC and 10 μL propidium iodide (PI) (20 μg/mL) were added and incubated in the dark for 15 min at room temperature. Finally, 400 μL binding buffer was added to each reaction tube before the cells were analyzed by FACScan flow cytometry. The data were analyzed by WinMDI V2.9 software (The Scripps Research Institute, San Diego, CA, USA) [40].

### Cell-cycle analysis

Cell-cycle analysis was performed using the standard method [40] with some modifications. Briefly, 5×10^5^ cells/well were seeded in 6-well plates and starved in serum-free medium at 37°C. After 12 h starvation, the cells were treated with Huaier extract or/and tamoxifen for 48 h. The cells were then trypsinized, washed with cold PBS, and fixed overnight with 75% cold ethanol at -20°C. The next day, the fixed cells were washed with PBS twice. After that, the cell plates were suspended with 200 μL RNase A (1 mg/mL) at 37°C for 10 min, followed by the addition of 300 μL PI (100 μg/mL) to stain the DNA of cells in the dark. After 20 min incubation at room temperature, the DNA contents of the cells were analyzed with a FACScan flow cytometer and the data were analyzed by ModFitLT V2.0 software (Becton Dickinson).

### Terminal-deoxynucleotidyl transferase-mediated nick end labeling (TUNEL) assay

TUNEL staining was performed using the One Step TUNEL Apoptosis Assay Kit (Beyotime, Jiangsu, China) according to the manufacturer's instructions. After treatment, the cells werefixed with 4% paraformaldehyde phosphate buffered saline, rinsed with PBS, and then permeabilized with0.1% Triton X-100 for 2 min on ice followed by the application of the TUNEL kit for 1 h at 37°C. The TUNEL-positive cells (red fluorescence) were imaged using fluorescent microscopy.

### Western blot analysis

MCF-7 and T47D cells were treated with Huaier extract (2, 4, or 6 mg/mL) and tamoxifen (5, 10, or 20 μM) for 48 h. Proteins were collected from different cell groups lysed in lysis buffer in the presence of protease inhibitors [41]. Proteins were separated by 12% SDS-PAGE and electro-blotted onto a PVDF membrane using a semi-dry blotting apparatus (Bio-Rad, Hercules, CA, USA). After blocking with 5% nonfat milk, the membranes were incubated overnight at 4°C with the primary antibodies, followed by labeling with the secondary antibody. Protein bands were visualized using the Pro-lighting HRP agent. β-actin was used as the endogenous control.

### Xenograft tumorigenicity assay

Cells (5×10^6^ in 0.2 mL PBS) were injected subcutaneously into 4-week-old BALB/c nu/nu female mice (Taconic). For the xenograft tumorigencity assay in MCF-7 cells, a 17β-estradiol pellet (Innovative Research of America) was implanted into each mouse 5 days before injection. After 2 days, the mice were randomly assigned to vehicle control (tri-distilled water), Huaier extract alone, TAM alone, or combined treatment groups. The Huaier group was given a 100 μL solution containing 50 mg while the TAM dosage was 5mg/kg. Drugs were administered by gavage once every three days. Tumor growth was measured every 5 days and tumor volume was calculated using the following equation: Volume = (width^2^×length) /2. After 40 days, the mice were sacrificed and the xenografts were removed for immunohistochemical staining and western blot assays.

### Immunohistochemical analysis

Immunohistochemistry was performed as described previously [42, 43]. After excision, the tumor tissues were stored in 10% neutral-buffered formalin. After 24 h, the samples were paraffin-embedded and sliced into 4 μm sections. The sections were microwaved for antigen retrieval and incubated with primary antibody overnight at 4°C. Then the sections were washed with PBS, treated with biotinylated anti-immunoglobulin antibody for 20 min, and allowed to react with horseradish peroxidase-conjugated streptavidin. Following detection with diaminobenzidine, the sections were counterstained with hematoxylin. The representative images of tumor tissues were taken using an Olympus light microscope.

### Statistical analysis

SPSS software (version 18.0) was used for statistical analysis. Student's *t*-tests and one-way ANOVAs were performed to determine significance. All error bars represent the standard error of the mean (SEM) of three experiments, and differences with *p* < 0.05 were considered significant.

## References

[1] Burstein HJ, Temin S, Anderson H, Buchholz TA, Davidson NE, Gelmon KE, Giordano SH, Hudis CA, Rowden D, Solky AJ, Stearns V, Winer EP, Griggs JJ (2014). Adjuvant endocrine therapy for women with hormone receptor-positive breast cancer: american society of clinical oncology clinical practice guideline focused update. J Clin Oncol.

[2] Sawka CA, Pritchard KI, Shelley W, DeBoer G, Paterson AH, Meakin JW, Sutherland DJ (1997). A randomized crossover trial of tamoxifen versus ovarian ablation for metastatic breast cancer in premenopausal women: a report of the National Cancer Institute of Canada Clinical Trials Group (NCIC CTG) trial MA. 1. Breast cancer research and treatment.

[3] Vogel VG, Costantino JP, Wickerham DL, Cronin WM, Cecchini RS, Atkins JN, Bevers TB, Fehrenbacher L, Pajon ER, Wade JL, Robidoux A, Margolese RG, James J, Lippman SM, Runowicz CD, Ganz PA (2006). Effects of tamoxifen vs raloxifene on the risk of developing invasive breast cancer and other disease outcomes: the NSABP Study of Tamoxifen and Raloxifene (STAR) P-2 trial. JAMA.

[4] Fisher B, Costantino JP, Wickerham DL, Redmond CK, Kavanah M, Cronin WM, Vogel V, Robidoux A, Dimitrov N, Atkins J, Daly M, Wieand S, Tan-Chiu E, Ford L, Wolmark N (1998). Tamoxifen for prevention of breast cancer: report of the National Surgical Adjuvant Breast and Bowel Project P-1 Study. J Natl Cancer Inst.

[5] Gorin MB, Day R, Costantino JP, Fisher B, Redmond CK, Wickerham L, Gomolin JE, Margolese RG, Mathen MK, Bowman DM, Kaufman DI, Dimitrov NV, Singerman LJ, Bornstein R, Wolmark N (1998). Long-term tamoxifen citrate use and potential ocular toxicity. Am J Ophthalmol.

[6] Tamoxifen for early breast cancer: an overview of the randomised trials (1998). Early Breast Cancer Trialists’ Collaborative Group. Lancet.

[7] Li C, Zhou C, Wang S, Feng Y, Lin W, Lin S, Wang Y, Huang H, Liu P, Mu YG, Shen X (2011). Sensitization of glioma cells to tamoxifen-induced apoptosis by Pl3-kinase inhibitor through the GSK-3beta/beta-catenin signaling pathway. PloS one.

[8] McCulloch M, See C, Shu XJ, Broffman M, Kramer A, Fan WY, Gao J, Lieb W, Shieh K, Colford JM (2006). Astragalus-based Chinese herbs and platinum-based chemotherapy for advanced non-small-cell lung cancer: meta-analysis of randomized trials. Journal of clinical oncology : official journal of the American Society of Clinical Oncology.

[9] Zhang N, Kong X, Yan S, Yuan C, Yang Q (2010). Huaier aqueous extract inhibits proliferation of breast cancer cells by inducing apoptosis. Cancer Sci.

[10] Wu T, Chen W, Liu S, Lu H, Wang H, Kong D, Huang X, Kong Q, Ning Y, Lu Z (2014). Huaier suppresses proliferation and induces apoptosis in human pulmonary cancer cells via upregulation of miR-26b-5p. FEBS Lett.

[11] Yan X, Lyu T, Jia N, Yu Y, Hua K, Feng W (2013). Huaier aqueous extract inhibits ovarian cancer cell motility via the AKT/GSK3beta/beta-catenin pathway. PloS one.

[12] Yang Q (2012). Anti-angiogenic and antitumor activities of Huaier aqueous extract. Oncology reports.

[13] Wang X, Qi W, Li Y, Zhang N, Dong L, Sun M, Cun J, Zhang Y, Lv S, Yang Q (2015). Huaier Extract Induces Autophagic Cell Death by Inhibiting the mTOR/S6K Pathway in Breast Cancer Cells. PloS one.

[14] Sun Y, Sun T, Wang F, Zhang J, Li C, Chen X, Li Q, Sun S (2013). A polysaccharide from the fungi of Huaier exhibits anti-tumor potential and immunomodulatory effects. Carbohydr Polym.

[15] Zheng J, Li C, Wu X, Liu M, Sun X, Yang Y, Hao M, Sheng S, Sun Y, Zhang H, Long J, Liang Y, Hu C (2014). Astrocyte elevated gene-1 (AEG-1) shRNA sensitizes Huaier polysaccharide (HP)-induced anti-metastatic potency via inactivating downstream P13K/Akt pathway as well as augmenting cell-mediated immune response. Tumour Biol.

[16] Pandiella1 A, Morís F, Ocaña A, Núñez N, Montero1 C (2015). Antitumoral activity of the mithralog EC-8042 in triple negative breast cancer linked to cell cycle arrest in G2. Oncotarget.

[17] Kilker RL, Planas-Silva MD (2006). Cyclin D1 is necessary for tamoxifen-induced cell cycle progression in human breast cancer cells. Cancer research.

[18] Chueh-Chuan Yen Y-CL, Wang, Chen, Chung-Der Hsiao, Ting-Wei Chang, Jen-Hwey Chiu, Paul Chih-Hsueh Chen, Wei-Ming Chen, Fang-Yi Yao, Hsei-Wei Wang, Chien-Lin Liu, Shih-Chieh Hung, Chi-Hung Lin, Yao-Shan Wen, Jir-You, Cheng-Hwai Tzeng, Tain-Hsiung, Jonathan A (2014). Fletcher Cytotoxic effects of 15d-PGJ2 against osteosarcoma through ROS-mediated AKT and cell cycle inhibition. oncotarget.

[19] Liao1 K, Juan L, Zhiling W (2014). Dihydroartemisinin inhibits cell proliferation via AKT/GSK3β/cyclinD1 pathway and induces apoptosis in A549 lung cancer cells. Int J Clin Exp Pathol.

[20] Karlsson E, Veenstra C, Emin S, Dutta C, Perez-Tenorio G, Nordenskjold B, Fornander T, Stal O (2015). Loss of protein tyrosine phosphatase, non-receptor type 2 is associated with activation of AKT and tamoxifen resistance in breast cancer. Breast cancer research and treatment.

[21] Vivanco I, Sawyers CL (2002). The phosphatidylinositol 3-Kinase AKT pathway in human cancer. Nat Rev Cancer.

[22] Perez-Tenorio G, Stal O (2002). Activation of AKT/PKB in breast cancer predicts a worse outcome among endocrine treated patients. Br J Cancer.

[23] Wang X, Zhang N, Huo Q, Sun M, Lv S, Yang Q (2013). Huaier aqueous extract suppresses human breast cancer cell proliferation through inhibition of estrogen receptor alpha signaling. Int J Oncol.

[24] Boya P, Gonzalez-Polo RA, Casares N, Perfettini JL, Dessen P, Larochette N, Metivier D, Meley D, Souquere S, Yoshimori T, Pierron G, Codogno P, Kroemer G (2005). Inhibition of macroautophagy triggers apoptosis. Mol Cell Biol.

[25] Barnard G, Hopkins L, Moorthie S, Seilly D, Tonks P, Dabaghian R, Clewley J, Coward J, McConnell I (2007). Direct detection of disease associated prions in brain and lymphoid tissue using antibodies recognizing the extreme N terminus of PrPC. Prion.

[26] Ojha R, Ishaq M, Singh SK (2015). Caspase-mediated crosstalk between autophagy and apoptosis: Mutual adjustment or matter of dominance. Journal of cancer research and therapeutics.

[27] Mizushima N, Levine B, Cuervo AM, Klionsky DJ (2008). Autophagy fights disease through cellular self-digestion. Nature.

[28] Dikic I, Johansen T, Kirkin V (2010). Selective autophagy in cancer development and therapy. Cancer research.

[29] Klionsky DJ, Emr SD (2000). Autophagy as a regulated pathway of cellular degradation. Science.

[30] Hengartner MO (2000). The biochemistry of apoptosis. natural.

[31] Quelle DE, Ashmun RA, Shurtleff SA, Kato JY, Bar-Sagi D, Roussel MF, Sherr CJ (1993). Overexpression of mouse D-type cyclins accelerates G1 phase in rodent fibroblasts. Genes & development.

[32] Brugge J, Hung MC, Mills GB (2007). A new mutational AKTivation in the PI3K pathway. Cancer Cell.

[33] Wang F, Mao Y, You Q, Hua D, Cai D (2015). Piperlongumine induces apoptosis and autophagy in human lung cancer cells through inhibition of PI3K/Akt/mTOR pathway. Int J Immunopathol Pharmacol.

[34] Levine B, Yuan J (2005). Autophagy in cell death: an innocent convict?. J Clin Invest.

[35] Vaux DL, Korsmeyer SJ (1999). Cell death in development. Cell.

[36] Chiu HW, Ho SY, Guo HR, Wang YJ (2009). Combination treatment with arsenic trioxide and irradiation enhances autophagic effects in U118-MG cells through increased mitotic arrest and regulation of PI3K/Akt and ERK1/2 signaling pathways. Autophagy.

[37] Nizamutdinova IT, Lee GW, Son KH, Jeon SJ, Kang SS, Kim YS, Lee JH, Seo HG, Chang KC, Kim HJ (2008). Tanshinone I effectively induces apoptosis in estrogen receptor-positive (MCF-7) and estrogen receptor-negative (MDA-MB-231) breast cancer cells. International journal of oncology.

[38] Sabol M, Trnski D, Uzarevic Z, Ozretic P, Musani V, Rafaj M, Cindric M, Levanat S (2014). Combination of cyclopamine and tamoxifen promotes survival and migration of mcf-7 breast cancer cells--interaction of Hedgehog-Gli and estrogen receptor signaling pathways. PloS one.

[39] Li LQ, Xie WJ, Pan D, Chen H, Zhang L (2015). Inhibition of autophagy by bafilomycin A1 promotes chemosensitivity of gastric cancer cells. Tumour Biol.

[40] Cheng YL, Chang WL, Lee SC, Liu YG, Chen CJ, Lin SZ, Tsai NM, Yu DS, Yen CY, Harn HJ (2004). Acetone extract of Angelica sinensis inhibits proliferation of human cancer cells via inducing cell cycle arrest and apoptosis. Life Sci.

[41] Zhou L, Chan WK, Xu N, Xiao K, Luo H, Luo KQ, Chang DC (2008). Tanshinone IIA, an isolated compound from Salvia miltiorrhiza Bunge, induces apoptosis in HeLa cells through mitotic arrest. Life Sci.

[42] Yang Q, Moran MS, Haffty BG (2009). Bcl-2 expression predicts local relapse for early-stage breast cancer receiving conserving surgery and radiotherapy. Breast Cancer Res Treat.

[43] Haffty BG, Yang Q, Reiss M, Kearney T, Higgins SA, Weidhaas J, Harris L, Hait W, Toppmeyer D (2006). Locoregional relapse and distant metastasis in conservatively managed triple negative early-stage breast cancer. J Clin Oncol.

